# Diagnostic performance of quantitative and qualitative elastography for the differentiation of benign and malignant cervical lymph nodes: A protocol for systematic review and meta-analysis

**DOI:** 10.1097/MD.0000000000027958

**Published:** 2021-11-24

**Authors:** Zhaohua Jia, Meijing Qu, Lipeng Sun, Hui Wang

**Affiliations:** Ultrasound Department of the First Affiliated Hospital of Dalian Medical University, China.

**Keywords:** lymph nodes, meta-analysis, shear wave elastography, strain elastography

## Abstract

**Background:**

Studies have shown inconsistent results regarding the diagnostic performance of quantitative and qualitative elastography for the differentiation of benign and malignant cervical lymph nodes. This meta-analysis aimed to estimate the diagnostic performance of ultrasound elastography in patients with cervical lymphadenopathy.

**Methods:**

We will search PubMed, Web of Science, Cochrane Library, and Chinese biomedical databases from their inceptions to the May 30, 2021, without language restrictions. Two authors will independently carry out searching literature records, scanning titles and abstracts, full texts, collecting data, and assessing risk of bias. Review Manager 5.2 and Stata14.0 software will be used for data analysis.

**Results:**

This systematic review will determine the accuracy of shear wave elastography and strain elastography in the differential diagnosis between benign and malignant cervical lymph nodes.

**Conclusion:**

Its findings will provide helpful evidence for the accuracy of shear wave elastography and strain elastography in the differential diagnosis between benign and malignant cervical lymph nodes.

**Systematic review registration:**

INPLASY202150109.

## 1 Introduction

As an important part of the body's immune system, lymph nodes are distributed in all parts of the body, and their changes are closely related to the corresponding drainage area lesions. The cervical lymph nodes account for about 40% of the total lymph nodes. Therefore, it is often regarded as an important symptom or staging basis of a variety of diseases, and is highly valued by clinicians. Early and accurate diagnosis of superficial lymph node lesions is of great significance for guiding treatment. In recent years, cytology and histopathology are considered as reliable means of examination, but they are invasive and can cause discomfort and anxiety in patients. Ultrasound is the first choice for the differential diagnosis of lymph nodes because of its convenience and efficiency.^[1–3]^ Ultrasound elastography is a noninvasive detection method that can reflect information about the stiffness of the lesion.^[4,5]^ According to different imaging principles, ultrasound elastography can be methodologically divided into quantitative and qualitative elastography to assess tissue stiffness.^[4,5]^ Quantitative elastography, mainly shear wave imaging, uses short-duration acoustic radiation forces to generate small localized tissue displacements (1–10 μm), which cause shear wave propagation and are tracked to calculate shear wave velocity or converted to Young modulus to reflect tissue stiffness. Qualitative elastography, mainly strain imaging, reflects tissue stiffness through the color gradation superimposed on grayscale ultrasound images.^[9]^ Based on the fact that malignant lesions are usually harder than normal tissue, many studies have explored the diagnostic value of ultrasound elastography for the differentiation of benign and malignant superficial cervical lymph nodes. However, there is a lack of large sample study on the diagnostic value of ultrasound elastography in cervical lymph nodes. So it is necessary to perform a meta-analysis to assess the diagnostic value of ultrasound elastography for the differentiation of benign and malignant cervical lymph nodes. Thus, to explore the impact of different imaging principles, we simultaneously investigated the diagnostic performance of quantitative and qualitative elastography for the differentiation of benign and malignant cervical lymph nodes in this meta-analysis.

## 2 Materials and methods

This study was conducted in accordance with the PRISMA (Preferred Reporting Items for Systematic Reviews and Meta-Analyses) guidelines and the protocol was registered in the INPLASY (INPLASY202150109).

### 2.1 Eligibility criteria

#### 2.1.1 Type of study

This study will only include high quality clinical cohort or case control studies.

#### 2.1.2 Type of patients

The patients should be those who with enlarged lymph nodes in the neck.

#### 2.1.3 Intervention and comparison

This study compare shear wave elastography (SWE) with strain elastography (SE) for diagnosing benign and malignant cervical lymph nodes.

#### 2.1.4 Type of outcomes

The primary outcomes include sensitivity, specificity, positive and negative likelihood ratio, diagnostic odds ratio, and the area under the curve of the summary receiver operating characteristic.

### 2.2 Search methods

PubMed, Web of Science, Cochrane Library, and Chinese biomedical databases will be searched from their inceptions to the May 30, 2021, without language restrictions. The search strategy for PubMed is shown in Table 1. Other online databases will be used in the same strategy.

**Table 1:** Search strategy sample of pubmed.

Number	Search terms
1	Lymph nodes or lymphadenectasis or lymphadenopathy
2	Shear wave elastography or strain elastography or SWE or SE
3	Pathology
4	and 1–3

### 2.3 Data extraction and quality assessment

Two authors will independently select the trials according to the inclusion criteria, and import into Endnote X9. Then remove duplicated or ineligible studies. Screen the titles, abstracts, and full texts of all literature to identify eligible studies. All essential data will be extracted using previously created data collection sheet by 2 independent authors. Discrepancies in data collection between 2 authors will be settled down through discussion with the help of another author. The following data will be extracted from each included research: the first author surname, publication year, language of publication, study design, sample size, number of lesions, source of the subjects, instrument, “gold standard,” and diagnostic accuracy. The true positives, true negatives, false positives, and false negatives in the fourfold (2 × 2) tables were also collected. Methodological quality was independently assessed by 2 researchers based on the quality assessment of studies of diagnostic accuracy studies (QUADAS) tool. The QUADAS criteria included 14 assessment items. Each of these items was scored as “yes” (2), “no” (0), or “unclear” (1). The QUADAS score ranged from 0 to 28, and a score ≥22 indicated good quality. Any disagreements between 2 investigators will be solved through discussion or consultation by a 3rd investigator.

### 2.4 Statistical analysis

The STATA version 14.0 (Stata Corp, College Station, TX) and Meta-Disc version 1.4 (Universidad Complutense, Madrid, Spain) software were used for meta-analysis. We calculated the pooled summary statistics for sensitivity, specificity, positive and negative likelihood ratio, and diagnostic odds ratio with their 95% confidence intervals. The summary receiver operating characteristic curve and corresponding area under the curve were obtained. The threshold effect was assessed using Spearman correlation coefficients. The Cochrans *Q*-statistic and *I* test were used to evaluate potential heterogeneity between studies. If significant heterogeneity was detected (*Q* test *P* < .05 or *I* test >50%), a random effects model or fixed effects model was used. We also performed sub group and meta-regression analyses to investigate potential sources of heterogeneity. To evaluate the influence of single studies on the overall estimate, a sensitivity analysis was performed. We conducted Begg funnel plots and Egger linear regression tests to investigate publication bias.

### 2.5 Ethics and dissemination

We will not obtain ethic documents because this study will be conducted based on the data of published literature. We expect to publish this study on a peer-reviewed journal.

## 3 Discussion

Cervical lymph node disease is a common disease type, and cervical lymph node enlargement is a common manifestation after cervical lymph node disease, which is divided into begain and malignant. There are some differences in the treatment of different lesions. Therefore, it is of great significance to make clear the actual lesion type of patients as soon as possible when making the treatment plan of the disease. At present, ultrasonography is a common method for clinical diagnosis of lymph node diseases. Although the sensitivity of two-dimensional ultrasound is high, but the specificity is poor, it can not effectively distinguish the benign and malignant lymph nodes, which is easy to cause early diagnosis errors. Ultrasound elastography is an important milestone in the development of ultrasound imaging diagnosis technology. According to the characteristics of uneven distribution of elastic coefficient in the body tissue, the differential diagnosis of benign and malignant lesions is carried out. The biomechanical characteristics of ultrasound elastography are more sensitive than that of two-dimensional ultrasound, which can be further evaluated by ultrasound elastography.^[6]^ Real-time(strain)elastography and SWE are the 2 most widely used elastographic techniques.^[7]^ The basic principle of SE is based on the benign and malignant of different tissues. By comparing the SR of lymph nodes and surrounding tissues, find out the point of the junction value to judge the benign and malignant lesions.^[8]^ Various quantitative elasticity measurements can be obtained with SWE, including maximum elasticity, mean elasticity, minimum elasticity, the standard deviation of the region of interest, and the ratio of elasticity of the lesion to the surrounding normal tissue.^[9]^ We performed this meta-analysis to investigate the diagnostic performance of quantitative and qualitative elastography for the differentiation of benign and malignant superficial lymph nodes. Elastography was found to have good diagnostic performance in diagnosing cervical lymphadenopathy. Both quantitative and qualitative elastography were demonstrated to have good clinical utility in the diagnosis of cervical lymphadenopathy. Quantitative and qualitative elastography have comparable performance in diagnosing cervical lymphadenopathy. In conclusion, both SWE and SE examination techniques have high diagnostic value for the differential diagnosis of cervical lymphadenopathy. In clinical practice, we should combine various techniques to improve the accuracy of diagnosis.

## Author contributions

**Conceptualization:** Lipeng Sun, Hui Wang.

**Data curation:** Zhaohua Jia, Meijing Qu.

**Methodology:** Zhaohua Jia, Meijing Qu.

**Writing – original draft:** Zhaohua Jia.

**Writing – review & editing:** Zhaohua Jia, Hui Wang.
